# Effectiveness and safety of apixaban vs warfarin among venous thromboembolism patients at high-risk of bleeding

**DOI:** 10.1371/journal.pone.0274969

**Published:** 2022-09-23

**Authors:** Alexander T. Cohen, Janvi Sah, Amol D. Dhamane, Theodore Lee, Lisa Rosenblatt, Patrick Hlavacek, Birol Emir, Allison Keshishian, Huseyin Yuce, Xuemei Luo

**Affiliations:** 1 Department of Hematological Medicine, Guy’s & St Thomas’ NHS Foundation Trust, King’s College London, London, United Kingdom; 2 STATinMED, LLC, Dallas, TX, United States of America; 3 Bristol Myers Squibb Company, Lawrenceville, NJ, United States of America; 4 Pfizer, New York, NY, United States of America; 5 New York City College of Technology, City University of New York, New York, NY, United States of America; 6 Pfizer, Groton, CT, United States of America; Sapienza - University of Rome, ITALY

## Abstract

This study evaluated effectiveness and safety of apixaban versus warfarin among venous thromboembolism patients at high-risk of bleeding (defined as having at least one of the following bleeding risk factors: ≥75 years; used antiplatelet, NSAIDs, or corticosteroids; had prior gastrointestinal bleeding or gastrointestinal-related conditions; late stage chronic kidney disease). Adult venous thromboembolism patients initiating apixaban or warfarin with ≥1 bleeding risk factor were identified from Medicare and four commercial claims databases in the United States. To balance characteristics between apixaban and warfarin patients, stabilized inverse probability treatment weighting was conducted. Cox proportional hazards models were used to estimate the risk of recurrent venous thromboembolism, major bleeding, and clinically relevant non-major bleeding. In total, 88,281 patients were identified. After inverse probability treatment weighting, the baseline patient characteristics were well-balanced between the two cohorts. Among venous thromboembolism patients at high-risk of bleeding, apixaban was associated with significantly lower risk of recurrent venous thromboembolism, major bleeding and clinically relevant non-major bleeding. No significant interactions were observed between treatment and number of risk factors on major bleeding and clinically relevant non-major bleeding or between treatment and type of bleeding risk factors on any of the outcomes. In conclusion, apixaban was associated with significantly lower risk of recurrent venous thromboembolism and bleeding among venous thromboembolism patients at high-risk of bleeding. Effects were generally consistent across subgroups of patients with different number or type of bleeding risk factors.

## 1. Introduction

Venous thromboembolism (VTE), consisting of deep vein thrombosis (DVT) and pulmonary embolism (PE), is the third most common etiology of vascular death after myocardial infarction and stroke with an annual incidence of up to 2 per 1000 cases in the general population [1]. Over the past decade, direct oral anticoagulants (DOACs) including apixaban, dabigatran, edoxaban, and rivaroxaban have been approved for the treatment of VTE [2, 3]. A network meta-analysis of randomized clinical trials and other retrospective studies reported similar efficacy but significantly lower risk of major bleeding among patients that initiated DOACs compared to warfarin [4].

Bleeding is a serious medical condition. For example, intracranial haemorrhage can be detrimental. Other types of bleeding such as GI bleeding is also associated with an increase in morbidity and mortality and can be exacerbated by the use of blood-thinning medications [5]. Studies have shown that demographic and clinical characteristics like older age, history of bleeding, peptic ulcer, active cancer, prior stroke, chronic renal or liver disease, antiplatelet use, and poor anticoagulant control are common risk factors associated with an increase in the risk of bleeding among patients on anticoagulant treatment [6–8].

Currently, there is limited real-world evidence regarding the use of anticoagulants among VTE patients who are at high risk of bleeding. Patients at high risk of bleeding include those with at least one bleeding risk factor (e.g, ≥75 years; used antiplatelet, NSAIDs, or corticosteroids; had prior gastrointestinal bleeding or gastrointestinal-related conditions; late stage chronic kidney disease) [6–8]. The objective of this retrospective cohort study was to evaluate the effectiveness and safety of apixaban versus warfarin among VTE patients who are at high risk of bleeding using a pooled patient population in the United States.

## 2. Materials and methods

### 2.1. Data source and patient selection

**Fig 1:**
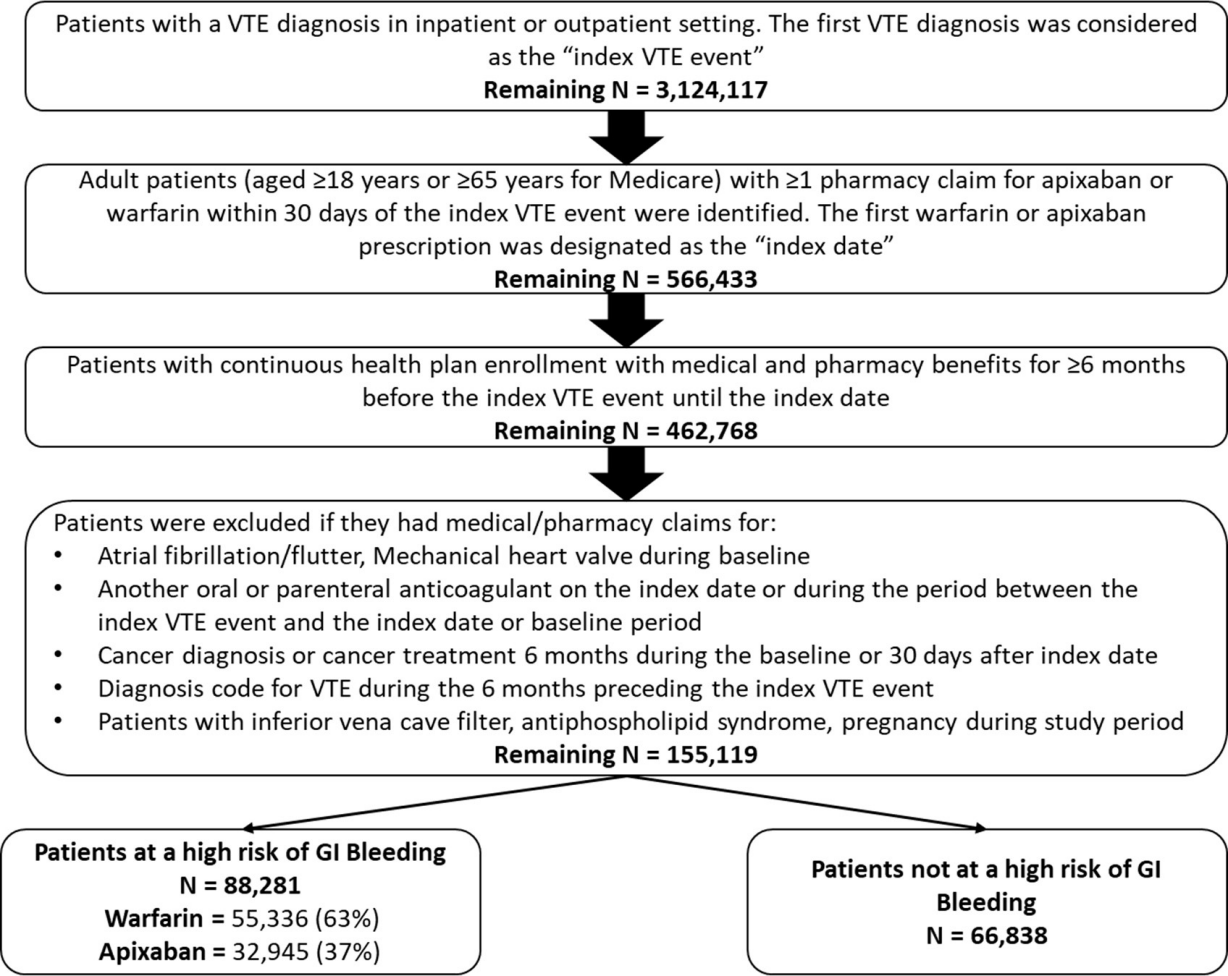
Inclusion criteria. VTE: venous thromboembolism.

Patients with ≥1 medical claim for VTE in any position (index VTE event) in the inpatient or outpatient setting were pooled from five databases: Centers for Medicare & Medicaid Services fee-for-service Medicare data (March 1, 2014 –December 31, 2017), IBM® MarketScan® Commercial Claims and Encounter and Medicare Supplemental and Coordination of Benefits Database (MarketScan: March 1, 2014 –September 30, 2018), IQVIA PharMetrics Plus™ (PharMetrics: March 1, 2014 –March 31, 2019), Optum Clinformatics™ Data Mart (Optum: March 1, 2014 –December 31, 2018), and the Humana® Research Database (Humana: March 1, 2014 –December 31, 2018). Adult patients (aged ≥18 years for commercial databases and ≥65 years for the Medicare database) were selected if they had ≥1 pharmacy claim for warfarin or apixaban during the 30-day period following the index VTE event. The first warfarin or apixaban prescription date was designated as the index date. Patients with active cancer (defined as ≥2 of the same cancer diagnosis or ≥1 cancer diagnosis and a cancer-related treatment [e.g., chemotherapy, radiation, cancer-related surgery] within 6 months before the index VTE event or 30 days after index VTE event were excluded. Additional selection criteria are listed in Fig 1. The baseline period was defined as 6 months prior to and including the index date and patients were followed from the day after the index date through the earliest of the following: end of the subsequent 6-month period; index therapy discontinuation; switch to another oral anticoagulant or parenteral anticoagulant treatment; health plan disenrollment; death; or study end.

VTE patients with a high risk of bleeding were defined as those who had at least one of the following risk factors: aged ≥75 years; used an antiplatelet, NSAID, or corticosteroid; had GI bleed or GI-related conditions [peptic ulcer, helicobacter pylori infection, diverticulosis, angiodysplasias, history of GI cancer (stomach, colon, esophageal, or rectal), or other GI lesions]; had later stage chronic kidney disease [CKD: stages III, IV or V/end stage renal disease (ESRD)]) [6, 7]. Patients with a high risk of bleeding were stratified based on the index treatment—apixaban vs warfarin.

### 2.2. Study measures

Demographics (age, sex, geographic region) and clinical characteristics (Deyo-Charlson comorbidity index [CCI], baseline comorbidities, falls, fracture/trauma involving lower extremities, selected surgeries, and baseline medications) were evaluated during the baseline period whereas setting of the index VTE event (inpatient vs outpatient), type of index VTE event (DVT only vs PE with or without DVT), and index VTE etiology (unprovoked vs provoked events—defined as index VTE event that were preceded within 3 months by hormone therapy, fracture/trauma involving lower extremities, pelvic/orthopedic surgery, or hospitalization for any reason for ≥3 days) were measured on the index VTE event date [9].

Clinical outcomes including recurrent VTE, MB, and CRNM bleeding were evaluated during the follow-up period among VTE patients with a high risk of bleeding and definitions of these outcomes followed previous publications [10–13]. Recurrent VTE was defined by an ICD-9/ICD-10 diagnosis for VTE in a primary/first-listed position in an inpatient setting; any inpatient VTE event occurring within 7 days of the index VTE event was excluded due to proximity to the index VTE event. MB was defined by an ICD-9/ICD-10 diagnosis or procedure code for GI, intracranial or other MB events in a primary/first-listed position in an inpatient setting. MB events were stratified by site of bleeding (GI, intracranial hemorrhage [ICH] and other sites [genitourinary bleeding, respiratory tract bleeding, ocular bleeding, joint bleeding/hemarthrosis, transfusion of blood and blood components, other bleeding, or no bleeding site specified]). CRNM bleeding was defined as noncritical site bleeding that did not qualify as MB but required either hospitalization with the bleeding as a secondary diagnosis or an outpatient visit. Specifically, CRNM bleeding events include either inpatient admission with a secondary diagnosis code for non-critical sites of bleeding or a diagnosis code for GI bleeding or other selected non-critical site of bleeding in the outpatient setting.

### 2.3. Statistical methods

Patient characteristics between the apixaban and warfarin cohorts were balanced using stabilized inverse probability treatment weighting (IPTW) within each database [14]. Covariates included in the model were demographics, clinical characteristics, and VTE-related variables; propensity scores were calculated using a logistic model with the two treatment cohorts. Each patient was weighted by the inverse of the probability of their treatment option, and the weights were stabilized by multiplying the original weights with a constant (expected value of being in the treatment or comparison cohorts, respectively) [15–17]. Balance of characteristics after IPTW was assessed using the standardized difference with a standardized difference >10 indicating significant differences and imbalance between groups.

After IPTW, the baseline characteristics of VTE patients with a high risk of bleeding were well balanced in each of the five databases, and patients were pooled for further analysis. Cox proportional hazard models, without taking into consideration of competing risk, were used to evaluate the risk of recurrent VTE, MB, and CRNM bleeding among VTE patients that initiated apixaban vs warfarin. Two interaction analyses were conducted to evaluate the treatment effect across the different subgroups: number of bleed risk factors (patients with only 1 risk factor, patients with 2 risk factors, and patients with 3 or more risk factors) and type of bleed risk factor among patients with only 1 risk factor (age ≥75, concomitant medication, prior GI bleeds or GI-related conditions, or CKD). The statistical significance (p<0.10) of the interaction between treatment and number of risk factors/type of risk factor on effectiveness and safety were evaluated.

## 3. Results

After applying the selection criteria, a total of 155,119 VTE patients were identified including 60,786 (39.2%) who initiated apixaban and 94,333 (60.8%) who initiated warfarin (**Fig 1**). Of the total VTE patients, 88,281 (56.9%) were categorized as having a high risk of bleeding and 66,838 (43.1%) patients not having a high risk of bleeding.

**Table 1** shows the baseline characteristics of VTE patients with or without a high risk of bleeding before IPTW. Patients with a high risk of bleeding were older (mean age: 74.2 vs 56.7), sicker (mean CCI: 3.0 vs 1.2), and more likely to be females (60.2% vs 47.6%) compared to VTE patients not at a high risk of bleeding. Additionally, patients with a high risk of bleeding were more likely to have an inpatient VTE diagnosis (61.2% vs 44.5%) and a provoked VTE (63.2% vs 47.0%) for the index VTE event compared to patients not at a high risk of bleeding. Patients with a high risk of bleeding were also more likely to have comorbidities such as hypertension (81.5% vs 52.2%), history of bleed (25.9% vs 13.6%), and hematologic disorders associated with bleeding (9.5% vs 6.0%).

**Table 1:** Descriptive baseline characteristics among VTE patients at and not at a high risk of bleeding.

	Patients at a high risk of bleeding	Patients not at a high risk of bleeding	STD^a^
**Sample Size**	**88,281**	**66,838**	** **
**Age in years, Mean (SD)**	74.2 (13.6)	56.7 (13.2)	130.51
**Sex, n (%)**			
Male	35,099 (39.8%)	35,012 (52.4%)	25.53
Female	53,182 (60.2%)	31,826 (47.6%)	25.53
**Setting of Index VTE Event, n (%)**			
Inpatient	54,066 (61.2%)	29,756 (44.5%)	33.98
Outpatient	34,215 (38.8%)	37,082 (55.5%)	33.98
**Index VTE Diagnosis, n (%)**			
Deep-vein thrombosis only	49,577 (56.2%)	38,039 (56.9%)	1.52
Pulmonary embolism with deep-vein thrombosis	38,704 (43.8%)	28,799 (43.1%)	1.52
Pulmonary embolism without deep-vein thrombosis	12,337 (14.0%)	9,638 (14.4%)	1.28
**Index VTE Etiology, n (%)**			
Provoked	55,801 (63.2%)	31,404 (47.0%)	33.06
Unprovoked	32,480 (36.8%)	35,434 (53.0%)	33.06
**Deyo-Charlson Comorbidity Index, Mean (SD)**	3.0 (2.5)	1.2 (1.6)	85.97
**Baseline Comorbidity, n (%)**			
Alcohol abuse	2,409 (2.7%)	2,474 (3.7%)	5.52
Anemia	33,729 (38.2%)	11,734 (17.6%)	47.32
Central venous Catheter	8,768 (9.9%)	3,794 (5.7%)	15.91
Hematologic disorders associated with bleeding^b^	8,380 (9.5%)	4,041 (6.0%)	12.90
Ischemic heart/ coronary artery disease	30,025 (34.0%)	9,225 (13.8%)	48.77
Dyspepsia or stomach discomfort	23,764 (26.9%)	11,582 (17.3%)	23.26
Hyperlipidemia	51,210 (58.0%)	23,348 (34.9%)	47.56
Obesity	23,893 (27.1%)	19,202 (28.7%)	3.71
Pneumonia	15,578 (17.6%)	6,780 (10.1%)	21.82
Rheumatologic disease	5,817 (6.6%)	1,454 (2.2%)	21.69
Sleep apnea	12,174 (13.8%)	8,616 (12.9%)	2.64
Spinal cord injury	212 (0.2%)	203 (0.3%)	1.22
Thrombophilia^c^	2,880 (3.3%)	3,210 (4.8%)	7.84
Varicose Veins	3,719 (4.2%)	2,852 (4.3%)	0.27
Hypertension	71,945 (81.5%)	34,864 (52.2%)	65.56
Chronic Kidney Disease—Stage I & II	3,501 (4.0%)	825 (1.2%)	17.23
Inflammatory Bowel Disease	2,045 (2.3%)	857 (1.3%)	7.79
History of Bleed	22,844 (25.9%)	9,084 (13.6%)	31.24
**Risk Factors for Bleed, n (%)**			
**Age ≥75 years (on index date)**	52,001 (58.9%)	n/a	n/a
**Concurrent medications (on index date)**	31,253 (35.4%)	n/a	n/a
Antiplatelets	7,932 (9.0%)	n/a	n/a
Nonsteroidal anti-inflammatory drug	14,247 (16.1%)	n/a	n/a
Corticosteroids	11,473 (13.0%)	n/a	n/a
**Prior GI conditions**	22,201 (25.1%)	n/a	n/a
Peptic ulcer	3,177 (3.6%)	n/a	n/a
Prior GI bleeding	8,340 (9.4%)	n/a	n/a
Helicobacter pylori	522 (0.6%)	n/a	n/a
Diverticulosis	13,810 (15.6%)	n/a	n/a
Angiodysplasias	257 (0.3%)	n/a	n/a
GI cancer	365 (0.4%)	n/a	n/a
Other GI lesions	1,260 (1.4%)	n/a	n/a
**Chronic Kidney Disease III-V/ESRD**	22,100 (25.0%)	n/a	n/a
Stage III	18,365 (20.8%)	n/a	n/a
Stage IV	5,080 (5.8%)	n/a	n/a
End Stage Renal Disease & Stage V	3,570 (4.0%)	n/a	n/a
**Number of Risk Factors for Bleeding** ^**d**^ **, n (%)**			
Only 1 risk factor	56,219 (63.7%)	n/a	n/a
2 risk factors	25,434 (28.8%)	n/a	n/a
3 risk factors	6,044 (6.8%)	n/a	n/a
4 risk factors	584 (0.7%)	n/a	n/a
**Index Medication**			
Apixaban	32,945 (37.3%)	27,841 (41.7%)	8.88
Warfarin	55,336 (62.7%)	38,997 (58.3%)	8.88

ESRD: end stage renal disease; GI: Gastrointestinal; IPTW: inverse probability treatment weighting; SD: Standard deviation; STD: standardized difference; VTE: venous thromboembolism

^a^ Standardized Difference = 100*|actual standardized difference|. Standardized Difference greater than 10 was considered significant.

^b^ Hematologic disorders associated with bleeding: conditions that hinder mediation of blood clotting and increase bleeding risk, e.g., Von Willebrand’s disease, the defibrination syndrome, acquired coagulation factor deficiency, unspecified coagulation defects, allergic purpura, qualitative platelet defects, nonthrombocytopenic purpuras, thrombocytopenia, and thrombotic microangiopathy.

^c^ Thrombophilia: conditions that increase the risk of blood clot development, e.g., diseases of blood and blood-forming organs, thalassemia, polycythemia vera, prothrombin gene mutation, and lupus anticoagulant syndrome.

^d^ Risk factors for bleeding include age≥75 years, used concomitant medication (antiplatelet, NSAIDs, or corticosteroids) on the index date, had a prior GI bleeds or GI-related conditions (peptic ulcer, helicobacter pylori infection, diverticulosis, angiodysplasias, GI cancer [stomach, colon, esophageal, or rectal], or other GI lesions), or had a diagnosis for chronic kidney disease (CKD: stages III, IV or V/end stage renal disease [ESRD]) during the baseline period.

**Table 2** represents the pre- and post-IPTW baseline characteristics among VTE patients with a high risk of bleeding who initiated apixaban vs warfarin. When stratified by treatment, 32,945 (37.3%) patients initiated apixaban and 55,336 (62.7%) initiated warfarin. Among patients with a high risk of bleeding, 58.9% were aged ≥75 years, 35.4% had concomitant medications, 25.1% had a prior GI condition, and 25.0% had CKD during baseline period. Additionally, 63.7% patients had only 1 risk factor, 28.8% had 2 risk factors, 6.8% had 3 risk factors, and 0.7% had all 4 risk factors.

**Table 2:** Descriptive pre- and post-IPTW baseline characteristics among VTE patients at a high risk of bleeding that initiated apixaban vs warfarin.

	Pre-IPTW			Post-IPTW^c^		
	**Warfarin Cohort (Reference)**	**Apixaban Cohort**	**STD** ^**a**^	**Warfarin Cohort (Reference)**	**Apixaban Cohort**	**STD** ^**a**^
**Sample Size**	55,336	32,945		55,336	32,945	
**Age in years, Mean (SD)**	74.5 (13.3)	73.6 (14.0)	6.12	74.6 (13.2)	73.4 (14.1)	8.39
**Age in years** ^**b**^ **, n (%)**						
18–54	4,785 (8.6%)	3,319 (10.1%)	4.90	4,636 (8.4%)	3,459 (10.5%)	7.26
55–64	4,830 (8.7%)	3,820 (11.6%)	9.50	4,943 (8.9%)	3,702 (11.2%)	7.65
65–74	12,622 (22.8%)	6,904 (21.0%)	4.48	12,407 (22.4%)	7,128 (21.6%)	1.90
75–79	12,064 (21.8%)	6,853 (20.8%)	2.44	12,052 (21.8%)	6,847 (20.8%)	2.43
≥80	21,035 (38.0%)	12,049 (36.6%)	2.98	21,298 (38.5%)	11,809 (35.8%)	5.47
**Sex** ^**b**^ **, n (%)**						
Male	21,756 (39.3%)	13,343 (40.5%)	2.42	21,721 (39.3%)	13,308 (40.4%)	2.33
Female	33,580 (60.7%)	19,602 (59.5%)	2.42	33,615 (60.7%)	19,637 (59.6%)	2.33
**Geographic Region** ^**b**^ **, n (%)**						
Northeast	8,882 (16.1%)	4,882 (14.8%)	3.41	8,789 (15.9%)	4,950 (15.0%)	2.38
North Central	17,058 (30.8%)	7,054 (21.4%)	21.56	15,236 (27.5%)	8,886 (27.0%)	1.27
South	18,593 (33.6%)	16,320 (49.5%)	32.77	21,603 (39.0%)	13,246 (40.2%)	2.38
West	10,696 (19.3%)	4,649 (14.1%)	14.02	9,614 (17.4%)	5,811 (17.6%)	0.70
Other	107 (0.2%)	40 (0.1%)	1.82	94 (0.2%)	53 (0.2%)	0.20
**Setting of Index VTE Event** ^**b**^ **, n (%)**						
Inpatient	34,940 (63.1%)	19,126 (58.1%)	10.42	34,169 (61.7%)	19,839 (60.2%)	3.14
Outpatient	20,396 (36.9%)	13,819 (41.9%)	10.42	21,167 (38.3%)	13,106 (39.8%)	3.14
**Index VTE Diagnosis** ^**b**^ **, n (%)**						
Deep-vein thrombosis only	31,308 (56.6%)	18,269 (55.5%)	2.27	31,003 (56.0%)	18,655 (56.6%)	1.20
Pulmonary embolism with deep-vein thrombosis	7,774 (14.0%)	4,563 (13.9%)	0.57	7,812 (14.1%)	4,522 (13.7%)	1.13
Pulmonary embolism without deep-vein thrombosis	16,254 (29.4%)	10,113 (30.7%)	2.89	16,521 (29.9%)	9,768 (29.6%)	0.45
**Index VTE Etiology** ^**b**^ **, n (%)**						
Provoked	36,214 (65.4%)	19,587 (59.5%)	12.39	35,494 (64.1%)	20,416 (62.0%)	4.50
Unprovoked	19,122 (34.6%)	13,358 (40.5%)	12.39	19,842 (35.9%)	12,529 (38.0%)	4.50
**Follow-up, days, Mean (SD)**	127.8 (60.5)	119.4 (60.2)		127.7 (60.7)	118.8 (60.2)	14.70
**Deyo-Charlson Comorbidity Index** ^**b**^ **, Mean (SD)**	3.0 (2.5)	2.9 (2.5)	4.41	3.0 (2.5)	3.0 (2.5)	1.68
**Baseline Comorbidity** ^**b**^ **, n (%)**						
Alcohol abuse	1,539 (2.8%)	870 (2.6%)	0.86	1,491 (2.7%)	945 (2.9%)	1.07
Anemia	22,087 (39.9%)	11,642 (35.3%)	9.46	21,345 (38.6%)	12,477 (37.9%)	1.44
Central venous catheter	5,927 (10.7%)	2,841 (8.6%)	7.07	5,443 (9.8%)	3,344 (10.2%)	1.05
Hematologic disorders associated with bleeding^d^	5,647 (10.2%)	2,733 (8.3%)	6.59	5,278 (9.5%)	3,177 (9.6%)	0.36
Ischemic heart/ coronary artery disease	18,773 (33.9%)	11,252 (34.2%)	0.48	19,023 (34.4%)	10,982 (33.3%)	2.20
Dyspepsia or stomach discomfort	15,066 (27.2%)	8,698 (26.4%)	1.86	14,865 (26.9%)	8,970 (27.2%)	0.82
Hyperlipidemia	31,883 (57.6%)	19,327 (58.7%)	2.12	32,345 (58.5%)	18,824 (57.1%)	2.66
Obesity	14,512 (26.2%)	9,381 (28.5%)	5.05	14,808 (26.8%)	9,048 (27.5%)	1.58
Pneumonia	9,817 (17.7%)	5,761 (17.5%)	0.67	9,830 (17.8%)	5,769 (17.5%)	0.67
Rheumatologic disease	3,714 (6.7%)	2,103 (6.4%)	1.33	3,688 (6.7%)	2,114 (6.4%)	1.01
Sleep apnea	7,577 (13.7%)	4,597 (14.0%)	0.76	7,601 (13.7%)	4,526 (13.7%)	0.01
Spinal cord injury	141 (0.3%)	71 (0.2%)	0.81	134 (0.2%)	82 (0.2%)	0.11
Thrombophilia^e^	1,877 (3.4%)	1,003 (3.0%)	1.97	1,785 (3.2%)	1,095 (3.3%)	0.55
Varicose veins	2,192 (4.0%)	1,527 (4.6%)	3.32	2,329 (4.2%)	1,364 (4.1%)	0.34
Hypertension	45,334 (81.9%)	26,611 (80.8%)	2.96	45,364 (82.0%)	26,616 (80.8%)	3.06
						
CKD Stage I & II	2,093 (3.8%)	1,408 (4.3%)	2.50	2,210 (4.0%)	1,322 (4.0%)	0.10
Inflammatory bowel disease	1,333 (2.4%)	712 (2.2%)	1.66	1,266 (2.3%)	777 (2.4%)	0.48
History of bleed	15,398 (27.8%)	7,446 (22.6%)	12.05	14,307 (25.9%)	8,615 (26.2%)	0.67
**Recent History of Falls** ^**b**^ **, n (%)**	5,183 (9.4%)	3,003 (9.1%)	0.87	5,209 (9.4%)	2,995 (9.1%)	1.11
**Fracture/trauma involving Lower Extremities** ^**b**^ **, n (%)**	8,519 (15.4%)	5,368 (16.3%)	2.46	8,761 (15.8%)	5,183 (15.7%)	0.28
**Selected Surgeries** ^**b**^ **, n (%)**	16,413 (29.7%)	9,073 (27.5%)	4.69	15,915 (28.8%)	9,633 (29.2%)	1.05
**Baseline Medication Use** ^**b**^ **, n (%)**						
Antiarrhythmic	6,412 (11.6%)	3,790 (11.5%)	0.26	6,367 (11.5%)	3,790 (11.5%)	0.01
Statins	25,860 (46.7%)	15,643 (47.5%)	1.50	26,148 (47.3%)	15,318 (46.5%)	1.52
Anti-platelets	6,675 (12.1%)	4,152 (12.6%)	1.64	6,813 (12.3%)	3,993 (12.1%)	0.59
Aromatase Inhibitors	127 (0.2%)	67 (0.2%)	0.56	122 (0.2%)	69 (0.2%)	0.26
Beta Blockers	24,374 (44.0%)	13,894 (42.2%)	3.78	24,159 (43.7%)	14,177 (43.0%)	1.27
Gastroprotective Agents	19,670 (35.5%)	11,403 (34.6%)	1.96	19,550 (35.3%)	11,525 (35.0%)	0.72
Selective Estrogen Receptor Modulator	398 (0.7%)	260 (0.8%)	0.81	423 (0.8%)	232 (0.7%)	0.71
Nonsteroidal Anti-inflammatory Drug	15,717 (28.4%)	10,748 (32.6%)	9.18	16,296 (29.4%)	10,130 (30.7%)	2.83
Hormone Therapy	1,735 (3.1%)	1,207 (3.7%)	2.92	1,805 (3.3%)	1,138 (3.5%)	1.06
**Risk Factors for Bleeding, n (%)**						
**Age ≥75 years (on index date)**	33,099 (59.8%)	18,902 (57.4%)	4.96	33,349 (60.3%)	18,657 (56.6%)	7.39
**Concurrent medications (on index date)** ^**b**^	18,843 (34.1%)	12,410 (37.7%)	7.55	19,285 (34.9%)	11,947 (36.3%)	2.95
Antiplatelets	4,858 (8.8%)	3,074 (9.3%)	1.92	4,958 (9.0%)	2,949 (9.0%)	0.03
Nonsteroidal anti-inflammatory drug	8,303 (15.0%)	5,944 (18.0%)	8.19	8,659 (15.6%)	5,572 (16.9%)	3.43
Corticosteroids	7,063 (12.8%)	4,410 (13.4%)	1.85	7,127 (12.9%)	4,369 (13.3%)	1.13
**Prior GI conditions** ^**b**^	14,160 (25.6%)	8,041 (24.4%)	2.73	13,842 (25.0%)	8,386 (25.5%)	1.01
Peptic ulcer	2,092 (3.8%)	1,085 (3.3%)	2.64	1,982 (3.6%)	1,185 (3.6%)	0.09
Prior GI bleeding	5,564 (10.1%)	2,776 (8.4%)	5.63	5,173 (9.3%)	3,186 (9.7%)	1.10
Helicobacter pylori	338 (0.6%)	184 (0.6%)	0.69	323 (0.6%)	196 (0.6%)	0.15
Diverticulosis	8,728 (15.8%)	5,082 (15.4%)	0.96	8,659 (15.6%)	5,164 (15.7%)	0.07
Angiodysplasias	183 (0.3%)	74 (0.2%)	2.02	161 (0.3%)	98 (0.3%)	0.15
GI cancer (Stomach, colon, esophageal, and rectal cancer)	242 (0.4%)	123 (0.4%)	1.01	232 (0.4%)	131 (0.4%)	0.35
Other GI lesions	696 (1.3%)	564 (1.7%)	3.76	784 (1.4%)	478 (1.5%)	0.28
**Chronic Kidney Disease III-V/ESRD** ^**b**^						
Stage III	11,572 (20.9%)	6,793 (20.6%)	0.72	11,573 (20.9%)	6,856 (20.8%)	0.25
Stage IV	3,570 (6.5%)	1,510 (4.6%)	8.19	3,208 (5.8%)	1,912 (5.8%)	0.02
End Stage Renal Disease & Stage V	2,666 (4.8%)	904 (2.7%)	10.89	2,236 (4.0%)	1,379 (4.2%)	0.74
**Number of Risk Factors for GI Bleed** ^**d**^ **, n (%)**						
Only 1 risk factor	34,855 (63.0%)	21,364 (64.8%)	3.87	34,832 (62.9%)	21,282 (64.6%)	3.44
2 risk factors	20,481 (37.0%)	11,581 (35.2%)	3.87	20,504 (37.1%)	11,663 (35.4%)	3.44
3 risk factors	3,882 (7.0%)	2,162 (6.6%)	1.80	3,866 (7.0%)	2,167 (6.6%)	1.62
4 risk factors	366 (0.7%)	218 (0.7%)	0.00	369 (0.7%)	210 (0.6%)	0.34

ESRD: end stage renal disease; GI: Gastrointestinal; IPTW: inverse probability treatment weighting; SD: Standard deviation; STD: standardized difference; VTE: venous thromboembolism

^a^ Standardized Difference = 100*|actual standardized difference|. Standardized Difference greater than 10 was considered significant.

^b^ Variables that were adjusted in inverse probability of treatment weighting

^c^ After applying weights, the values for categorical variables were not whole numbers; therefore, due to rounding the sum of patients may not equal 100%

^d^ Hematologic disorders associated with bleeding: conditions that hinder mediation of blood clotting and increase bleeding risk, e.g., Von Willebrand’s disease, the defibrination syndrome, acquired coagulation factor deficiency, unspecified coagulation defects, allergic purpura, qualitative platelet defects, nonthrombocytopenic purpuras, thrombocytopenia, and thrombotic microangiopathy.

^e^ Thrombophilia: conditions that increase the risk of blood clot development, e.g., diseases of blood and blood-forming organs, thalassemia, polycythemia vera, prothrombin gene mutation, and lupus anticoagulant syndrome.

^f^ Risk factors for bleeding include age≥75 years, used concomitant medication (antiplatelet, NSAIDs, or corticosteroids) on the index date, had a prior GI bleeds or GI-related conditions (peptic ulcer, helicobacter pylori infection, diverticulosis, angiodysplasias, GI cancer [stomach, colon, esophageal, or rectal], or other GI lesions), or had a diagnosis for chronic kidney disease (CKD: stages III, IV or V/end stage renal disease [ESRD]) during the baseline period.

**Fig 2:**
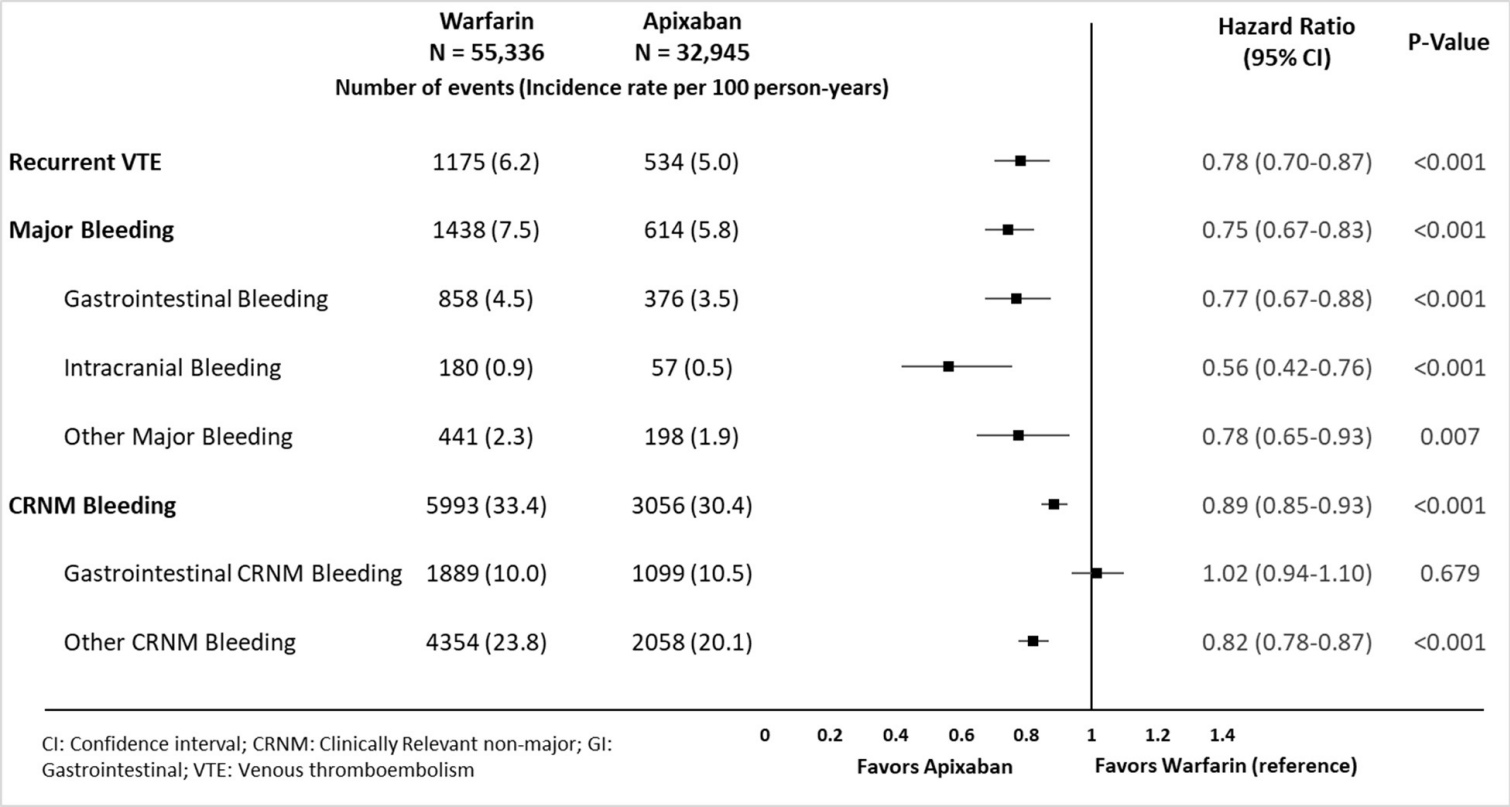
Risk of recurrent VTE, major bleeding, and CRNM bleeding for apixaban vs. warfarin among VTE patients at a high risk of bleeding.

After applying IPTW among patients with a high risk of bleeding, the baseline patient characteristics were balanced between apixaban and warfarin cohorts; mean age was ~74 years with a mean CCI score of 3.0. Follow-up time was comparable between warfarin (127.7 days) and apixaban (118.8 days) cohorts. Among VTE patients with a high risk of bleeding, apixaban was associated with a significantly lower risk of recurrent VTE (HR: 0.78; 95% CI: 0.70–0.87), overall MB (HR: 0.75; 95% CI: 0.67–0.83), gastrointestinal MB (HR: 0.77; 95% CI: 0.67–0.88), intracranial MB (HR: 0.56; 95% CI: 0.42–0.76), other MB (HR:0.78; 95% CI: 0.65–0.93), and CRNM bleeding (HR: 089; 95% CI: 0.85–0.93) compared to warfarin. But no significant difference was observed between apixaban and warfarin for the risk of GI CRNM bleeding (HR:1.02; 95% CI: 0.94–1.10; (**Fig 2**).

**Fig 3:**
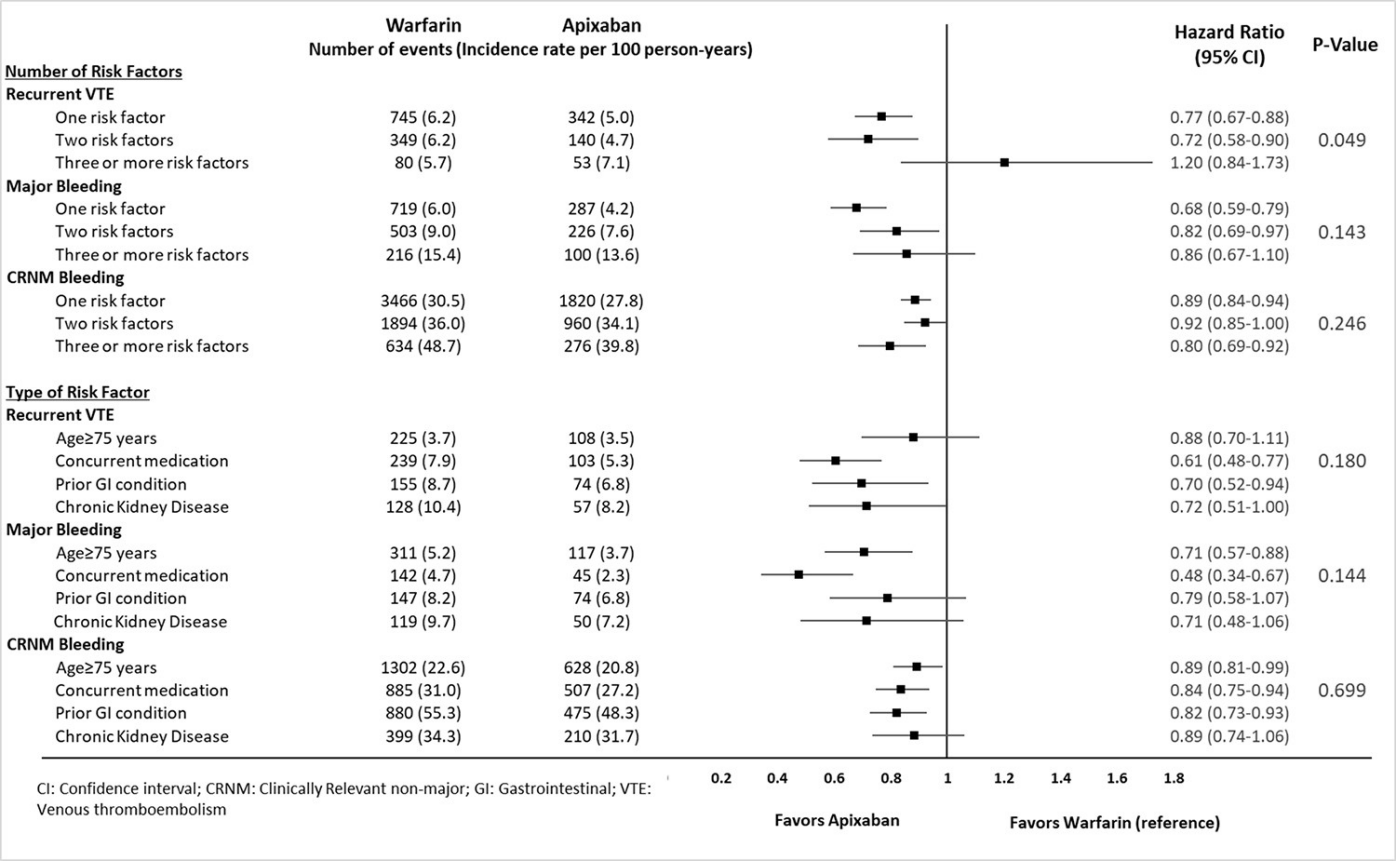
Risk of recurrent VTE, major bleeding, and CRNM bleeding for apixaban vs. warfarin among VTE patients at a high risk of bleeding stratified by number of risk factors and type of risk factors.

**S1 and S2 Tables** include baseline characteristics of VTE patients at high risk of bleeding stratified by the number of bleed risk factors and type of bleed risk factor, respectively. When stratified by number of bleed risk factors, no significant interactions were observed between treatment and the number of risk factors on MB (interaction p = 0.143) and CRNM bleeding (interaction p = 0.246; **Fig 3**). One significant interaction was observed between treatment and number of risk factors on recurrent VTE (interaction p = 0.049): apixaban showed a lower risk of recurrent VTE compared to warfarin in patients with 1 or 2 risk factors, whereas there was a trend toward a higher risk in the ~8% of patients with 3 or more risk factors. When stratified by type of bleed risk factor, no significant interactions were observed between treatment and type of risk factors on recurrent VTE (interaction p = 0.180), MB (interaction p = 0.144), and CRNM bleeding (interaction p = 0.699; **Fig 3**).

## 4. Discussion

In this real-world observational study pooling 5 US claims database, we found that among VTE patients who were at a high risk of bleeding, apixaban patients were associated with significantly lower risk of recurrent VTE, MB, and CRNM bleeding compared to warfarin patients. The treatment effects on MB and CRNMB were consistently observed across VTE patients with different number and different type of bleeding risk factors. Given that more than half of the patients in this study were found to be at a high risk for bleeding, our findings provide additional evidence to inform treatment options for these high-risk patients.

VTE patients at a high risk of bleeding present big challenges for clinical management. As found in this study, these patients tended to be older and have higher comorbid burden in addition to the higher risk of bleeding. While anticoagulant treatment has been found to prevent recurrence of VTE [18–21], there is a risk of bleeding associated with anticoagulants and the bleeding risk is further exacerbated in VTE patients at a high risk of bleeding. Evidence about the risk of recurrent VTE and bleeding associated with anticoagulant treatments will help inform appropriate treatment option for this vulnerable group of patients. But VTE patients at a high risk of bleed have been excluded or under-represented in randomized clinical trials. For example, the AMPLIFY trial excluded VTE patients at a high risk of bleeding [18]. Real-world Data studies can provide needed information on VTE patients at a high risk of bleed. The findings of this analysis of real-world data provided complementary evidence to the above randomized trial and showed that among patients at a risk of bleeding apixaban was associated with significantly lower risk of recurrent VTE, MB, and CRNM bleeding as well as major GI bleeding compared to warfarin.

Several real-world data studies have evaluated the effectiveness and safety of apixaban vs. warfarin among VTE patients with individual risk factors for bleeding such as old age, patients with CKD, or antiplatelet use [11, 22, 23]. In a Medicare analysis of VTE patients with an average age of 78 years, apixaban had a similar risk of recurrent VTE and significantly lower risk of MB and CRNM bleeding compared to warfarin [11]. In a retrospective multicenter chart review, the risk of thrombotic or bleeding events was lower in apixaban patients (HR: 0.47; 95% CI: 0.25–0.92) compared to warfarin patients with creatinine clearance <25 mL/min [22]. Additionally, Lutsey et al. reported that apixaban patients were associated with lower risk of hospitalized bleeding compared to warfarin (HR: 0.68; 95% CI: 0.50–0.92); no significant interaction was observed between treatment and kidney disease (interaction p = 0.14), but one significant interaction was observed between treatment and age (interaction p = 0.06) for hospitalized bleeding [23]. Our study extended these analyses and evaluated the effectiveness and safety of apixaban versus warfarin using a much more comprehensive list of risk factors for bleeding and including a much bigger sample of VTE patients at a high risk of bleeding. The findings of this analysis were generally consistent with that of the previous analyses and showed that apixaban patients were associated with significantly lower risk of recurrent VTE, MB, and CRNM bleeding as well as lower risk of major GI bleeding compared to warfarin patients among VTE patients at a high risk of bleed. Additionally, the mean follow-up time for the warfarin and apixaban cohorts in this study was comparable to that of prior studies [11, 22, 23]. The results of this analysis will help in addressing some of the safety concerns regarding anticoagulant use in this high risk patient population.

The present study has some limitations, most of which are inherent to claims databases. First, association can be inferred from the analysis, but causal relationships cannot be established. Second, there were no lab values collected; therefore, no data were available regarding international normalized ratio monitoring for warfarin, nor for laboratory assessment of renal impairment. Third, although IPTW has been used to balance patient characteristics between treatment cohorts, residual confounding may persist. Fourth, information on mortality was unavailable in some of the databases; hence, mortality or fatal VTE could not be evaluated. Patients who are at high risk of bleeding may also be at a higher risk of death and mortality which could be a competing risk in this analysis. Fifth, the use of over-the-counter medications such as aspirin which could increase the risk of bleeding could not be evaluated. Sixth, recurrent VTE and MB events were identified based on primary/first-listed ICD 9/10 diagnosis codes, and the number of events may be different compared with those identified in clinical trials which were based on adjudication. It is also possible that symptomatic events which were not diagnosed were not captured in the study. Additionally, CRNM bleeding definition has not been validated in the literature, but attempts were made to follow the definition as suggested by the International Society on Thrombosis and Haemostasis [24]. Finally, p value for interactions did not account for multiple subgroup analyses.

## 5. Conclusions

Among VTE patients at a high risk of bleeding, patients who initiated apixaban were associated with a significantly lower risk of recurrent VTE, overall MB including gastrointestinal MB, and CRNM bleeding as compared to patients who initiated treatment with warfarin. The treatment effects were generally consistent across subgroups of patients with different number and different type of risk factors. This real-world data study adds to the limited evidence and provides additional data to inform anticoagulant decision for VTE patients who are at a high risk of bleeding.

## Supporting information

S1 Table Post-IPTW descriptive baseline characteristics among VTE patients at a high risk of bleeding stratified by number of risk factors. (DOCX)

S2 Table Post-IPTW descriptive baseline characteristics among VTE patients at a high risk of bleeding stratified by type of risk factor. (DOCX)
