# Supplementary material for: Effectiveness and safety of apixaban vs warfarin among venous thromboembolism patients at high-risk of bleeding
Source: PLoS One. 2022 Sep 23;17(9):e0274969. doi: 10.1371/journal.pone.0274969 (PMC9506658; doi:10.1371/journal.pone.0274969)
Supplement: S2 Table — (DOCX) [file pone.0274969.s002.docx]

S2 Table. Post-IPTW descriptive baseline characteristics among VTE patients at a high risk of bleeding stratified by type of risk factor

|  | **Among patients with one risk factor for gastrointestinal bleed** | | | | | | | | | | | |
| --- | --- | --- | --- | --- | --- | --- | --- | --- | --- | --- | --- | --- |
|  | **Age ≥75 years** | | | **Concurrent medications** | | | **Prior GI conditions** | | | **Chronic Kidney Disease** | | |
|  | **Warfarin Cohort (Reference)** | **Apixaban Cohort** | **STD^a^** | **Warfarin Cohort (Reference)** | **Apixaban Cohort** | **STD^a^** | **Warfarin Cohort (Reference)** | **Apixaban Cohort** | **STD^a^** | **Warfarin Cohort (Reference)** | **Apixaban Cohort** | **STD^a^** |
| **Sample Size** | 16,842 | 9,451 |  | 9,081 | 6,086 |  | 5,276 | 3,476 |  | 3,633 | 2,269 |  |
| **Age in years, Mean (SD)** | 82.7 (6.3) | 82.8 (6.6) | 1.42 | 60.0 (12.1) | 58.8 (11.7) | 9.61 | 61.8 (11.0) | 60.5 (11.9) | 10.85 | 65.2 (8.8) | 64.9 (9.5) | 4.23 |
| **Gender, n (%)** |  |  |  |  |  |  |  |  |  |  |  |  |
| Male | 5,500 (32.7%) | 3,164 (33.5%) | 1.76 | 4,323 (47.6%) | 2,940 (48.3%) | 1.41 | 2,598 (49.2%) | 1,668 (48.0%) | 2.51 | 1,838 (50.6%) | 1,225 (54.0%) | 6.72 |
| Female | 11,342 (67.3%) | 6,287 (66.5%) | 1.76 | 4,758 (52.4%) | 3,146 (51.7%) | 1.41 | 2,678 (50.8%) | 1,809 (52.0%) | 2.51 | 1,794 (49.4%) | 1,044 (46.0%) | 6.72 |
| **Setting of Index VTE Event, n (%)** |  |  |  |  |  |  |  |  |  |  |  |  |
| Inpatient | 9,581 (56.9%) | 5,309 (56.2%) | 1.44 | 4,694 (51.7%) | 3,137 (51.5%) | 0.29 | 3,286 (62.3%) | 2,166 (62.3%) | 0.07 | 2,502 (68.9%) | 1,463 (64.5%) | 9.22 |
| Outpatient | 7,261 (43.1%) | 4,142 (43.8%) | 1.44 | 4,387 (48.3%) | 2,949 (48.5%) | 0.29 | 1,990 (37.7%) | 1,310 (37.7%) | 0.07 | 1,131 (31.1%) | 806 (35.5%) | 9.22 |
| **Index VTE Diagnosis, n (%)** |  |  |  |  |  |  |  |  |  |  |  |  |
| Deep-vein thrombosis only | 9,650 (57.3%) | 5,428 (57.4%) | 0.27 | 5,053 (55.6%) | 3,324 (54.6%) | 2.04 | 2,700 (51.2%) | 1,775 (51.1%) | 0.22 | 2,126 (58.5%) | 1,405 (61.9%) | 6.80 |
| Pulmonary embolism with deep-vein thrombosis | 2,269 (13.5%) | 1,288 (13.6%) | 0.46 | 1,155 (12.7%) | 813 (13.4%) | 1.93 | 865 (16.4%) | 542 (15.6%) | 2.14 | 541 (14.9%) | 281 (12.4%) | 7.25 |
| Pulmonary embolism without deep-vein thrombosis | 4,923 (29.2%) | 2,735 (28.9%) | 0.64 | 2,874 (31.7%) | 1,948 (32.0%) | 0.78 | 1,712 (32.4%) | 1,159 (33.3%) | 1.90 | 965 (26.6%) | 583 (25.7%) | 1.92 |
| **Index VTE Etiology, n (%)** |  |  |  |  |  |  |  |  |  |  |  |  |
| Provoked | 10,063 (59.7%) | 5,496 (58.2%) | 3.24 | 5,016 (55.2%) | 3,254 (53.5%) | 3.55 | 3,309 (62.7%) | 2,129 (61.3%) | 3.00 | 2,417 (66.5%) | 1,430 (63.0%) | 7.28 |
| Unprovoked | 6,779 (40.3%) | 3,954 (41.8%) | 3.24 | 4,065 (44.8%) | 2,832 (46.5%) | 3.55 | 1,967 (37.3%) | 1,347 (38.7%) | 3.00 | 1,215 (33.5%) | 839 (37.0%) | 7.28 |
| **Deyo-Charlson Comorbidity Index, Mean (SD)** | 2.1 (2.0) | 2.1 (2.0) | 0.35 | 1.8 (2.0) | 1.7 (1.9) | 5.51 | 2.0 (2.1) | 2.0 (2.2) | 2.55 | 5.2 (2.2) | 5.0 (2.4) | 6.59 |
| **Baseline Comorbidity, n (%)** |  |  |  |  |  |  |  |  |  |  |  |  |
| Alcohol abuse | 226 (1.3%) | 164 (1.7%) | 3.16 | 309 (3.4%) | 199 (3.3%) | 0.72 | 379 (7.2%) | 236 (6.8%) | 1.59 | 118 (3.2%) | 79 (3.5%) | 1.30 |
| Anemia | 4,954 (29.4%) | 2,759 (29.2%) | 0.49 | 2,141 (23.6%) | 1,395 (22.9%) | 1.56 | 2,101 (39.8%) | 1,374 (39.5%) | 0.61 | 1,903 (52.4%) | 1,122 (49.5%) | 5.80 |
| Central venous Catheter | 665 (3.9%) | 391 (4.1%) | 0.97 | 699 (7.7%) | 506 (8.3%) | 2.27 | 854 (16.2%) | 550 (15.8%) | 1.03 | 759 (20.9%) | 443 (19.5%) | 3.40 |
| Hematologic disorders associated with bleeding^b^ | 1,218 (7.2%) | 684 (7.2%) | 0.03 | 657 (7.2%) | 418 (6.9%) | 1.44 | 618 (11.7%) | 350 (10.1%) | 5.26 | 483 (13.3%) | 297 (13.1%) | 0.59 |
| Ischemic heart/ coronary artery disease | 4,957 (29.4%) | 2,796 (29.6%) | 0.35 | 2,348 (25.9%) | 1,540 (25.3%) | 1.28 | 1,196 (22.7%) | 722 (20.8%) | 4.60 | 1,371 (37.7%) | 819 (36.1%) | 3.37 |
| Dyspepsia or stomach discomfort | 2,892 (17.2%) | 1,599 (16.9%) | 0.66 | 1,683 (18.5%) | 1,124 (18.5%) | 0.16 | 2,778 (52.6%) | 1,831 (52.7%) | 0.05 | 837 (23.0%) | 537 (23.6%) | 1.44 |
| Hyperlipidemia | 9,225 (54.8%) | 5,231 (55.4%) | 1.17 | 4,304 (47.4%) | 2,823 (46.4%) | 2.02 | 2,504 (47.5%) | 1,535 (44.1%) | 6.64 | 2,489 (68.5%) | 1,471 (64.8%) | 7.75 |
| Obesity | 2,715 (16.1%) | 1,541 (16.3%) | 0.51 | 3,125 (34.4%) | 2,131 (35.0%) | 1.27 | 1,836 (34.8%) | 1,191 (34.3%) | 1.10 | 1,642 (45.2%) | 1,007 (44.4%) | 1.67 |
| Pneumonia | 2,469 (14.7%) | 1,347 (14.3%) | 1.16 | 1,387 (15.3%) | 955 (15.7%) | 1.18 | 826 (15.6%) | 565 (16.3%) | 1.64 | 692 (19.1%) | 406 (17.9%) | 2.94 |
| Rheumatologic disease | 680 (4.0%) | 335 (3.5%) | 2.62 | 799 (8.8%) | 497 (8.2%) | 2.28 | 194 (3.7%) | 143 (4.1%) | 2.33 | 126 (3.5%) | 93 (4.1%) | 3.26 |
| Sleep apnea | 1,351 (8.0%) | 738 (7.8%) | 0.78 | 1,618 (17.8%) | 1,071 (17.6%) | 0.55 | 907 (17.2%) | 597 (17.2%) | 0.06 | 859 (23.6%) | 501 (22.1%) | 3.68 |
| Spinal cord injury | 32 (0.2%) | 19 (0.2%) | 0.32 | 27 (0.3%) | 13 (0.2%) | 1.65 | 14 (0.3%) | 16 (0.5%) | 3.10 | 14 (0.4%) | 7 (0.3%) | 1.80 |
| Thrombophilia^c^ | 349 (2.1%) | 218 (2.3%) | 1.61 | 380 (4.2%) | 248 (4.1%) | 0.54 | 273 (5.2%) | 170 (4.9%) | 1.28 | 147 (4.0%) | 96 (4.2%) | 0.97 |
| Varicose Veins | 732 (4.3%) | 415 (4.4%) | 0.25 | 379 (4.2%) | 256 (4.2%) | 0.14 | 194 (3.7%) | 128 (3.7%) | 0.04 | 144 (4.0%) | 79 (3.5%) | 2.53 |
| Hypertension | 13,615 (80.8%) | 7,635 (80.8%) | 0.13 | 6,105 (67.2%) | 3,984 (65.5%) | 3.75 | 3,570 (67.7%) | 2,248 (64.7%) | 6.33 | 3,436 (94.6%) | 2,116 (93.3%) | 5.50 |
| Non-ESRD Renal Disease - Stage I & II | 464 (2.8%) | 250 (2.6%) | 0.69 | 152 (1.7%) | 99 (1.6%) | 0.37 | 97 (1.8%) | 77 (2.2%) | 2.65 | 330 (9.1%) | 206 (9.1%) | 0.09 |
| Inflammatory Bowel Disease | 158 (0.9%) | 78 (0.8%) | 1.19 | 189 (2.1%) | 130 (2.1%) | 0.36 | 261 (4.9%) | 172 (5.0%) | 0.07 | 57 (1.6%) | 36 (1.6%) | 0.21 |
| History of Bleed | 2,721 (16.2%) | 1,465 (15.5%) | 1.80 | 1,462 (16.1%) | 974 (16.0%) | 0.26 | 2,696 (51.1%) | 1,769 (50.9%) | 0.43 | 784 (21.6%) | 516 (22.7%) | 2.69 |

ESRD: end stage renal disease; GI: Gastrointestinal; IPTW: inverse probability treatment weighting; SD: Standard deviation; STD: standardized difference; VTE: venous thromboembolism

^a^ Standardized Difference=100*|actual standardized difference|. Standardized Difference greater than 10 was considered significant.

^b^ Hematologic disorders associated with bleeding: conditions that hinder mediation of blood clotting and increase bleeding risk, e.g., Von Willebrand’s disease, the defibrination syndrome, acquired coagulation factor deficiency, unspecified coagulation defects, allergic purpura, qualitative platelet defects, nonthrombocytopenic purpuras, thrombocytopenia, and thrombotic microangiopathy.

^c^ Thrombophilia: conditions that increase the risk of blood clot development, e.g., diseases of blood and blood-forming organs, thalassemia, polycythemia vera, prothrombin gene mutation, and lupus anticoagulant syndrome.
